# Case Report: Clinical Features of Childhood Leukoencephalopathy With Cerebral Calcifications and Cysts Due to *SNORD118* Variants

**DOI:** 10.3389/fneur.2021.585606

**Published:** 2021-06-17

**Authors:** Hong Jin, Xiaotun Ren, Husheng Wu, Yanqi Hou, Fang Fang

**Affiliations:** ^1^Department of Neurology, Beijing Children's Hospital, Capital Medical University, National Center for Children's Health, Beijing, China; ^2^Running Gene Inc., Beijing, China

**Keywords:** leukoencephalopathy, calcifications, cysts, *SNORD118*, child

## Abstract

**Background:** Leukoencephalopathy with cerebral calcifications and cysts (LCC) is a rare autosomal recessive cerebral microangiopathy. Recently, biallelic variants in a non-protein-coding gene *SNORD118* have been discovered to cause LCC.

**Case Presentation:** We here report a genetically confirmed childhood case of LCC. The patient was a 4-year-and-1-month-old boy with focal seizures. The age at onset of his seizure was 10 days after birth. The seizures were well-controlled by antiepileptic treatment but reoccurred twice due to a head impact accident and a fever, respectively. He suffered from a self-limited esotropia and unsteady running gait during the seizure onset. He had the typical neuroimaging triad of multifocal intracranial calcifications, cysts, and leukoencephalopathy. Genetic analysis indicated that he carried compound heterozygous variants of n.^*^9C>T and n.3C>T in *SNORD118*, which were inherited from his parents.

**Conclusion:** We report a childhood LCC case with compound heterozygous variants in *SNORD118*. To the best of our knowledge, the patient reported in our case had the youngest onset age of LCC with a determined genotype. The triad cerebral-imaging findings of calcifications, cysts, and leukoencephalopathy provide a crucial diagnostic basis. Moreover, the gene assessment, together with the clinical investigations, should be considered for the diagnosis of LCC.

## Introduction

Leukoencephalopathy with cerebral calcifications and cysts (LCC), first reported by Labrune in 1996, is a rare neurological microangiopathy characterized by leukoencephalopathy, intracranial calcifications, and cysts identified in cerebral imaging (1). About 100 cases have been reported so far (2–6). Leukoencephalopathy with cerebral calcifications and cysts has various clinical presentations, including seizures, cognitive decline, pyramidal/extrapyramidal signs, ataxia, and symptoms and signs consequent to increased intracranial pressure with the expanding cystic lesions (1, 7–10). Biallelic variants in gene *SNORD118* have been recently identified to be the cause of LCC (11). *SNORD118* is a non-protein-coding gene producing a special type of small nucleolar RNA (snoRNA), the box C/D snoRNA U8, which is involved in the modification and processing of rRNAs and the construction of ribosomes. It was investigated that disease-causing variations of *SNORD118* would affect the activity of U8, while the further pathological mechanism of the disease was still unclear (12). We here present a clinically and genetically confirmed childhood case of LCC in order to consolidate the understanding of clinical features and diagnostic bases of the disease.

## Case Presentation

**Figure 1:**
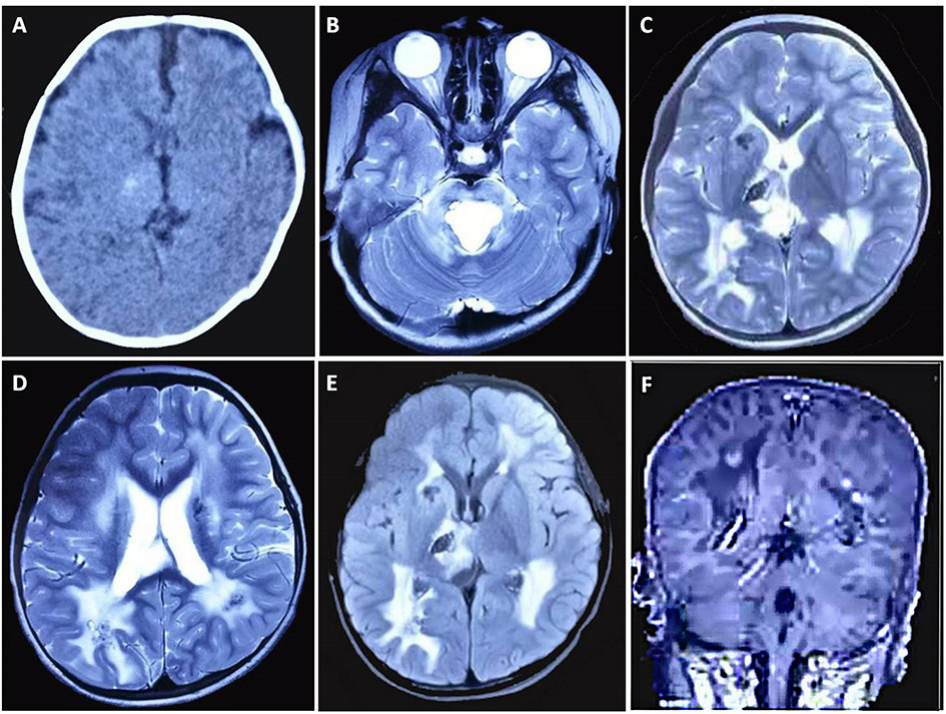
Brain CT and MRI findings of the case. **(A)** CT scan taken at 1 month after birth shows a focal calcification in the right thalamus. MRI [**(B–D)** T2WI, **(E)** FLAIR, **(F)** contrast-enhanced sequence] taken at 4 years and 1 month of age demonstrates bilateral calcifications and cysts, some with contrast enhancement, in the periventricular regions, basal ganglia, right thalamus, and pons, along with increased T2/FLAIR signal changes in periventricular white matter.

A 4-year-and-1-month-old boy was referred to us for intermittent focal seizures. The first onset of his seizure was at 10 days after birth, and his initial brain CT taken at 1 month of age showed a focal calcification in the right thalamus (Figure 1A). The seizures had been well-controlled by levetiracetam treatment (15 mg/kg/day, divided Q12h as an initial dose and increased gradually to 25 mg/kg/day, divided Q12h) for nearly 4 years but frequently recurred a week ago after he fell from a slide and had his head bump against the ground. He was born from unrelated parents after an uneventful pregnancy and developed normally. He could talk and walk independently at the age of 1 year. When he was 3 years old, he received the Gesell Institute's developmental examination with the DQ score of 92. No abnormality was found on general physical, ophthalmological, neurological, or biochemical blood examinations.

Magnetic resonance imaging demonstrated bilateral calcifications and cysts in the periventricular regions, basal ganglia, right thalamus, and pons. Some areas also had contrast enhancement. Increased T2/FLAIR (fluid-attenuated inversion recovery) signal was also identified in periventricular white matter (Figures 1B–F). According to the results described above, the patient was clinically diagnosed with LCC.

Whole-exome sequencing (WES) and Sanger sequencing were performed by MyGenostics (Beijing, China) using their standard process, which is available in the previous report (13). Compound heterozygous n.^*^9C>T and n.3C>T in *SNORD118* (NR_033294) of the patient were detected by genetic testing. Both two variants were inherited from his parents and had already been reported to cause disease (PP5) (11, 12). n.^*^9C>T is a paternal mutation in the region immediately downstream of *SNORD118*, while n.3C>T is a single-nucleotide substitution at the 5′ end of *SNORD118* inherited from his mother. They all have low allele frequencies in the genome databases of healthy controls [including 1,000 Genomes, ExAC, and gnomAD (PM2)] and could disturb the processing of *SNORD118* transcript (PVS1) (11, 14). Therefore, these two variants considered a likely pathogenic variant according to the standard of ACMG (15).

**Figure 2:**
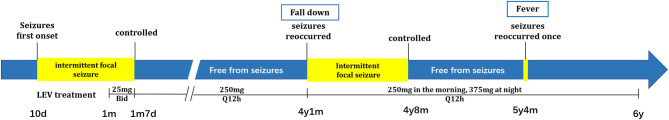
Patient's timeline of seizures and the following treatment. Patient's seizures periods were highlighted in yellow. The dosages of LEV treatment are shown under the diagram of disease onset. // was used to omit the time intervals that have no changes in clinical display or treatment.

The boy continued to receive oral antiepileptic treatment with levetiracetam (250 mg in the morning and 375 mg at night, Q12h, about 28 mg/kg/day) (Figure 2). In the following 2 years and 3 months, he suffered from a self-limited esotropia and unsteady running gait for 5 months, and his seizures were controlled after another 2 months. He was free from seizure for 8 months until he had a fever of 40°C at the age of 5 years and 4 months. His febrile seizures lasted for a few minutes and stopped without additional treatment, and no recurrence was noticed. The repeat cranial MRI did not show any notable changes. Moreover, he could run and jump freely, and learn things quickly in the kindergarten, as informed in a recent telephone follow-up.

## Discussion

Leukoencephalopathy with cerebral calcifications and cysts is a rare progressive chronic cerebral microangiopathy with various clinical presentations. So far, the clinical diagnosis of LCC was mainly counting on the neuroimaging screening. Characteristic neuroimaging features of LCC include asymmetric calcifications, diffuse leukoencephalopathy, multiple cysts of different sizes with contrast enhancement in the wall, and unusual bleeding in parenchyma or cysts (8–10, 16). Calcifications are reported predominantly in the basal ganglia, thalami, brainstem, dentate nuclei, and white matter, but may also be detected in the cerebral and cerebellar cortex (16, 17). With sparing of U fibers, corpus callosum, and gray matter, asymmetrical or relatively symmetrical leukoencephalopathy is usually distributed in periventricular and deep white matter, but relatively rare in the subcortical white matter. Multiple cysts can be observed over the entire brain parenchyma, especially appearing in a high frequency of the supratentorial region (2, 17). Susceptibility-weighted imaging of patients often exhibits micro-bleedings and micro-calcifications, which implies that LCC might be associated with the abnormalities of micro-vessels. Histopathological staining of biopsies from LCC patients can also be used for diagnosis. Angiomatous-like rearrangement of micro-vessels can always be identified as the primary prominent pathological feature, together with perivascular foci of dystrophic calcifications and hemosiderin deposits, hyaline degeneration, gliosis, and formation of Rosenthal fibers (1, 2, 8, 9). However, LCC was easy to be misdiagnosed as cerebroretinal microangiopathy with calcifications and cysts, which is also called Coats plus syndrome and appears similar neuroimaging displays and histopathological features, caused by variants of the *CTC1* gene (18). Our patient had the typical cerebral-imaging features of LCC, and the diagnosis was finally confirmed by genetic analysis. Since his initial CT scan taken at 1 month after birth revealed a focal calcification lesion in the right thalamus, we suspect that the disease might occur in the fetus and have already developed for a while.

*SNORD118* on chromosome 17p13.1 encodes the box C/D snoRNA U8, which is an evolutionarily conserved RNA involved in the biogenesis and function of ribosome (19). The snoRNA U8 consists of a conserved box C/D motif and LSm (like-Sm) binding site, which are important for the binding of four proteins (15.5K, NOP56, NOP58, and fibrillarin) and the formation of a ribonucleoprotein complex, respectively (20, 21). Variants in these two regions would result in reduced binding to the 15.5K protein and decrease the stability of the structure of the ribonucleoprotein complex in comparison to wild-type sequence (11), acting as functional null alleles. Otherwise, it has also been found that variants in the 5′ end and 3′ extension of U8 could disturb processing of the precursor U8 RNAs, but with a preserved function in ribosome biogenesis, acting as hypomorphic functional alleles (11, 14). A recently reported vertebrate U8 mutant animal model of LCC-associated U8 variants confirmed that the combination of one severe (null) and one milder (hypomorphic) mutation was responsible for the occurrence of LCC disease (14). However, in our case, the compound heterozygous variants n.^*^9C>T and n.3C>T in *SNORD118* were detected, which both have been predicted as “mild” alleles. It seemed that the combination of these two variants presumably would not result in disease, as homozygous mutations were found in gnomAD (Table 1). It was predicted that such hypomorphic mutant homozygotes would result in the reduction of U8 activity to a level, which is compatible with initially normal development, but insufficient to maintain cellular homeostasis long-term (22). More cases with combination of two “mild” alleles or hypomorphic mutant homozygotes were needed for a better understanding of the molecular pathology of LCC.

**Table 1:** SNORD118 variants identified in patients with LCC.

**Variant^b^**	**Genomic coordinates^a^**	**gnomAD total allele count**	**Hom**	**Het**	**Calculated gnomAD frequency**
n.-70_*76del	g.8076696_8076977del				Novel
n.-54_-49del	g.8076955_8076960del	224,458	0	0	Novel
n.-7_22dup	g.8076885_8076913dup	231,956	0	0	Novel
n.-6G>A	g.8076912C>T	262,340	0	224	0.0008539
n.2T>C	g.8076905A>G	230,692	0	5	0.00002167
n.3C>A	g.8076904G>T	231,102	0	1	0.000004327
n.3C>T	g.8076904G>A	262,476	2	389	0.001482
n.5T>C	g.8076902A>G	231,322	0	11	0.0000475528
n.8G>A	g.8076899C>T	262,710	4	724	0.002756
n.8G>C	g.8076899C>G	262,710	0	4	0.00001523
n.19C>G	g. 8076888G>C	263,190	0	9	0.00003420
n.20C>T	g.8076887G>A	231,710	0	7	0.00003021
n.24C>A	g.8076883G>T	231,996	0	2	0.000008622
n.24C>T	g.8076883G>A	263,352	0	84	0.0003190
n.39G>T	g.8076868C>A	232,004	0	2	0.000008621
n.39G>C	g.8076868C>G	263,390	0	33	0.0001253
n.39_40insT	g.8076867_8076868insA	231,998	1	25	0.000108
n.42G>A	g.8076865C>T	263,436	0	282	0.001070
n.56dup	g.8076851dup	232,100	0	2	0.000008617
n.57G>T	g.8076850C>A	263,492	0	0	Novel
n.57G>A	g.8076850C>T	263,492	0	7	0.00002657
n.58dup	g.8076849dup	263,490	0	0	Novel
n.58A>G	g.8076849T>C	263,490	0	6	0.00002277
n.59T>G	g.8076848A>C	263,468	0	0	Novel
n.60G>C	g.8076847C>G	232,080	0	0	Novel
n.60_61insT	g.8076846_8076847insA	263,474	0	2	0.000007591
n.61A>G	g.8076846T>C	263,474	0	16	0.00006073
n.61A>T	g.8076846T>A	232,094	0	2	0.000008617
n.64G>A	g.8076843C>T	263,458	0	35	0.0001328
n.72A>G	g.8076835 T>C	232,094	0	16	0.00006894
n.73T>G	g.8076834A>C	232,092	0	0	Novel
n.74G>A	g.8076833C>T	263,478	0	16	0.00006073
n.74G>T	g.8076833C>A	263,478	0	0	Novel
n.75A>G	g.8076832T>C	232,094	0	4	0.00001723
n.75A>C	g.8076832T>G	31,392	0	1	0.00003186
n.81G>A	g.8076826C>T	232,082	0	11	0.00004740
n.81G>C	g.8076826C>G	232,082	0	0	Novel
n.82A>G	g.8076825T>C	263,450	0	18	0.00006832
n.92C>T	g.8076794G>A	263,358	0	11	0.00004177
n.100T>G	g.8076807A>C	232,038	0	0	Novel
n.103G>A	g.8076804C>T	232,002	0	4	0.00001724
n.104G>A	g.8076803C>T	263,390	0	110	0.0004176
n.113C>T	g.8076794G>A	263,358	0	11	0.00004177
n.117C>G	g.8076790G>C	263,344	0	17	0.0000646
n.118T>G	g.8076789A>C	231,904	0	1	0.000004312
n.119G>T	g.8076788C>A	263,272	0	0	Novel
n.126C>T	g.8076781G>A	263,122	0	31	0.0001178
n.127C>G	g.8076780G>C	231,700	0	10	0.00004316
n.130T>C	g.8076777A>G	231,804	0	2	0.000008628
n.131C>A	g.8076776G>T	263,148	0	0	Novel
n.131C>G	g.8076776G>C	263,148	0	4	0.00001520
n.131C>T	g.8076776G>A	263,148	0	32	0.0.000122
n.*1C>T	g.8076770G>A	rs117595965	5	1328	0.005054
n.*5C>G	g.8076766G>C	262,552	0	159	0.0006056
n.*9C>T	g.8076762G>A	262,616	2	505	0.001923
n.*10G>T	g.8076761C>A	262,502	4	655	0.002495
n.*10G>A	g.8076761C>T	262,502	0	30	0.000114

*gnomAD, genome aggregation database; Hom, homozygous; Het, heterozygous*.

*The asterisk denotes that the nucleotide in question is located in the 3' extension of human pre-U8*.

a *All genomic coordinates should be preceded by Chr17(GRCh37)*.

b *SNORD118 NR_033294.1*.

Abnormally processed rRNA would impact the construction of ribosomes, affect translation procedure, and might lead to the formation of abnormal cell lumps. It has been hypothesized that diffuse angiomatous dysplastic microangiopathy induces the hypoxia of nearby tissue and ultimately leads to the development of calcifications and cysts. Hemorrhage may also play a critical role in the formation and expansion of cysts. Additionally, abnormal translation-induced pathological myelination, demyelination, and edema in the tissue surrounding cysts may be jointly responsible for the leukoencephalopathy (5, 8, 9, 23). Based on the neuroimaging and histopathological staining of LCC patients, it seems that patients' clinical presentations depend on the location and severity of lesions, including the size of their calcification and cyst, and the severity of their leukoencephalopathy. This disease may break out early or progress rapidly in some patients within several years due to cerebral bleeding, while it may also remain stable in some patients for years due to individual differences (8).

The onset age of LCC patients with a determined genotype had been reported ranging from 3 weeks to 67 years old. The patient who had the earliest onset age was a female reported by Crow et al. (22), presenting seizures at the disease onset. In this manuscript, our case had his first seizure at 10 days after birth, which is earlier than her and all other reported cases until now. In addition, the symptoms of our case were much milder and simpler. The seizures reoccurred for multiple times but have all been controlled. Esotropia and unsteady gait were also developed but later released. At the current stage, the patient was well-developed without additional abnormalities being noticed. Clinical features of our case show that the onset age of LCC was not directly associated with the disease severity. Our case also suggested that for LCC patients, early treatment may result in a better prognosis. Patients that displayed squint during the disease onset were rare, and we report the first patient who had self-limited esotropia. In all reported cases, there have been another three patients harboring the n.3C>T variant and nine patients harboring the n.^*^9C>T variant, which were identified in our patients. Their brain CTs/MRIs were similar, while the clinical features were not identical. Two patients who harbored n.3C>T variants had gait problems, while the other two patients had spasticity (Table 2). Patients harboring the n.^*^9C>T variants showed seizers (4/10), developmental delay (2/10), intellectual disability (ID, 2/10), ataxia (1/10), gait problems (1/10), dystonic quadriplegia (1/10), progressive motor deficit (1/10), and idiopathic Parkinson's disease (1/10). We also noticed that for reported patients with n.^*^9C>T variation, male patients have a significantly earlier onset age (10 days, 4 months, and 6 months) than female patients (one for infancy, two for teens, and four for 3–50 years) (Table 2) (12). More cases are needed to confirm this conjecture.

**Table 2:** Clinical features of our patient and reported patients with the same disease-causing variant.

	**Our case**	**Iwama et al. (12)**	**Jenkinson et al. (11)**	**Jenkinson et al. (11)**	**Jenkinson et al. (11)**	**Jenkinson et al. (11)**	**Jenkinson et al. (11)**	**Jenkinson et al. (11)**	**Jenkinson et al. (11)**	**Jenkinson et al. (11)**	**Jenkinson et al. (11)**	**Osman et al. (3)**	**Osman et al. (3)**
		**(patient 4)**	**(F309)**	**(F1445)**	**(F691)**	**(F819_1)**	**(F819_2)**	**(F1127)**	**(F1172)**	**(F1288)**	**(F551)**	**(patient 2)**	**(patient 5)**
Variant	n.*9C>T	n.3C>T	n.3C>T	n.3C>T	n.*9C>T	n.*9C>T	n.*9C>T	n.*9C>T	n.*9C>T	n.*9C>T	n.*9C>T	n.*9C>T	n.*9C>T
	n.3C>T	n.19C>G	n.131C>G	n.81G>A	n.58A>G	n.*1C>T	n.*1C>T	n.100T>G	n.131C>G	n.59T>G	n.127C>G	n.74G>T	n.131C>T
Origin	Chinese	Japanese	North American	British	British	British	British	French	German	North American	Belgium	French	French
Gender	Male	Male	Female	Female	Male	Female	Female	Male	Female	Female	Female	Female	Female
Age at onset	10 days	N.D.	2 years	10 years	<4 months	Teens	Teens	<6 months	50 years	3 years	Infancy	28 years	47 years
Current age	6 years	N.D.	22 years	10 years	4 years	35 years	32 years	2 years	54 years	17 years	Died at age 28 years	38 years	60 years
Brain CT/MRI	Focal calcification in the right thalamus. Bilateral calcifications and cysts in the periventricular regions, basal ganglia, right thalamus, and pons.	Brain calcifications, leukoencephalopathy, intracranial cyst.	Multiple cysts and calcifications.	Multiple cysts and calcifications.	Multiple cysts and calcifications.	Multiple cysts and calcifications.	Multiple cysts and calcifications.	Multiple cysts and calcifications.	Multiple cysts and calcifications.	Multiple cysts and calcifications.	Multiple cysts and calcifications.	Severe bilateral and asymmetric white-matter lesions, extensive calcifications, and multiple cysts with ring enhancement of their wall	Multiple cysts with one voluminous cyst located in the right cerebellum. Bilateral and asymmetric white-matter lesions with many calcifications.
Clinical features	Seizure, esotropia, unsteady running gait.	Spasticity. (Other information not provided.)	Failure to achieve motor milestones, Progressive spasticity and dystonia with complete loss of speech.	Gait problems, probably slowly progressive motor deterioration with learning difficulties.	Severe developmental delay.	Epileptic seizures, some intellectual delay with hemiparesis.	Epileptic seizures, some intellectual delay with hemiparesis.	Moderate developmental delay.	Minor degree of ataxia and dysarthria.	Gait problems, mainly unilateral dystonia/spasticity.	Dystonic quadriplegia, progressive neurological decline.	Progressive motor deficit and epileptic seizures.	Idiopathic Parkinson's disease

*N.D., no data*.

Currently, symptomatic treatment is the primary therapy of LCC. Anti-epileptic therapy, corticosteroid therapy, and surgical techniques such as cystic puncture, cystic resection, and cystoventriculoperitoneal shunt may temporarily relieve the symptoms (2, 3, 6). Fay et al. (24) reported an LCC case who got a marked clinical improvement by biweekly infusions of bevacizumab, an inhibitor of vascular endothelial growth factor, for more than 1 year. This case suggests a promising solution, but efficacy and safety of this treatment still need to be confirmed by further studies.

## Conclusion

We report a childhood LCC case with compound heterozygous variants in *SNORD118*, who had the youngest onset age of LCC with a determined genotype, advancing the range of onset age to 10 days after birth. The triad neuroimaging findings of cerebral calcifications, cysts, and leukoencephalopathy are essential to the diagnosis of LCC. As biopsy is invasive and usually uneasily available, the combined usage of gene assessment and clinical identification should be considered for the diagnosis of LCC.

## Data Availability Statement

The datasets presented in this study can be found in online repositories. The names of the repository/repositories and accession number(s) can be found below: https://www.ncbi.nlm.nih.gov/, PRJNA647511.

## Ethics Statement

Written informed consent have already been provided to the parents of these patients, and they all agreed to publish the patient's disease information.

## Author Contributions

The project was designed by XR and supervised by XR and FF. HJ collected the data, investigated the results, and drafted the manuscript. HW assisted the clinical data curation and patients' care. YH performed the genetic analysis and revised the manuscript. FF also reviewed the manuscript. All authors contributed to the article and approved the submitted version.

## Conflict of Interest

YH was employed by the company Running Gene Inc. The remaining authors declare that the research was conducted in the absence of any commercial or financial relationships that could be construed as a potential conflict of interest.

## Abbreviations

LCC: leukoencephalopathy with cerebral calcifications and cysts
MRI: magnetic resonance imaging
CT: computed tomography
FLAIR: fluid-attenuated inversion recovery
snoRNA: small nucleolar RNA.
